# Machine learning and lean six sigma for targeted patient-specific quality assurance of volumetric modulated arc therapy plans

**DOI:** 10.1016/j.phro.2024.100617

**Published:** 2024-08-09

**Authors:** Nicola Lambri, Damiano Dei, Giulia Goretti, Leonardo Crespi, Ricardo Coimbra Brioso, Marco Pelizzoli, Sara Parabicoli, Andrea Bresolin, Pasqualina Gallo, Francesco La Fauci, Francesca Lobefalo, Lucia Paganini, Giacomo Reggiori, Daniele Loiacono, Ciro Franzese, Stefano Tomatis, Marta Scorsetti, Pietro Mancosu

**Affiliations:** a IRCCS Humanitas Research Hospital, Radiotherapy and Radiosurgery Department, via Manzoni 56, 20089 Rozzano, Milan, Italy; b Department of Biomedical Sciences, Humanitas University, via Rita Levi Montalcini 4, 20072 Pieve Emanuele, Milan, Italy; c IRCCS Humanitas Research Hospital, Quality Department, via Manzoni 56, 20089 Rozzano, Milan, Italy; d Dipartimento di Elettronica, Informazione e Bioingegneria, Politecnico di Milano, 20133 Milan, Italy; e Health Data Science Centre, Human Technopole, 20157 Milan, Italy; f Dipartimento di Fisica “Aldo Pontremoli”, Università degli Studi di Milano, Milan, Italy

**Keywords:** Machine learning, Lean six sigma, Quality, Radiotherapy, Clinical risk management

## Abstract

**Background and purpose:**

Radiotherapy plans with excessive complexity exhibit higher uncertainties and worse patient-specific quality assurance (PSQA) results, while the workload of measurement-based PSQA can impact the efficiency of the radiotherapy workflow. Machine Learning (ML) and Lean Six Sigma, a process optimization method, were implemented to adopt a targeted PSQA approach, aiming to reduce workload, risk of failures, and monitor complexity.

**Materials and methods:**

Lean Six Sigma was applied using DMAIC (define, measure, analyze, improve, and control) steps. Ten complexity metrics were computed for 69,811 volumetric modulated arc therapy (VMAT) arcs from 28,612 plans delivered in our Institute (2013–2021). Outlier complexities were defined as >95th-percentile of the historical distributions, stratified by treatment. An ML model was trained to predict the gamma passing rate (GPR-3 %/1mm) of an arc given its complexity. A decision support system was developed to monitor the complexity and expected GPR. Plans at risk of PSQA failure, either extremely complex or with average GPR <90 %, were identified. The tool’s impact was assessed after nine months of clinical use.

**Results:**

Among 1722 VMAT plans monitored prospectively, 29 (1.7 %) were found at risk of failure. Planners reacted by performing PSQA measurement and re-optimizing the plan. Occurrences of outlier complexities remained stable within 5 %. The expected GPR increased from a median of 97.4 % to 98.2 % (Mann-Whitney p < 0.05) due to plan re-optimization.

**Conclusions:**

ML and Lean Six Sigma have been implemented in clinical practice enabling a targeted measurement-based PSQA approach for plans at risk of failure to improve overall quality and patient safety.

## Highlights

• A Machine Learning model highlighted plans with expected low gamma passing rate.
• Complexity and expected gamma were monitored prospectively with Lean Six Sigma.
• A Poka Yoke system automatically identified plans at risk of failure each day.
• Plans considered at risk underwent measurement and were re-optimized if necessary.
• Among 1722 volumetric modulated plans, 9 out of 29 at risk were actual failures.

## 1 Introduction

Currently, most radiotherapy (RT) plans are delivered via intensity-modulated techniques. The American Association of Physicists in Medicine Task Group (AAPM TG) 218 recommended that all modulated RT plans should undergo pre-treatment verification through a patient-specific quality assurance (PSQA) program [1]. PSQA is based on measurements to evaluate the agreement between the dose calculated by the treatment planning system (TPS) and the dose measured at the linear accelerator (linac). The agreement is quantified through a score, called gamma passing rate (GPR), which considers both the dose difference and the physical distance between the measured and calculated dose distributions [2].

The substantial workload associated with measurement-based PSQA can impact the efficiency of the RT workflow, potentially delaying the start of clinical treatments. The volume of intensity-modulated treatments has increased considerably in recent years and conducting individual PSQA measurements for every single case has become challenging [3]. At the same time, due to advancements in technology, improved planning techniques, and quality control, PSQA failures have become increasingly rare incidents [4], [5]. Therefore, to ensure that patient safety is maintained while preventing an excessive workload for the clinical staff, it is crucial to maximize the overall efficiency of the RT procedure and adopt a targeted PSQA approach.

To address this, plan complexity metrics have been proposed as predictors of the PSQA outcome, aiming to reduce the QA workload [6], [7]. Plan complexity attempts to quantify the uncertainties that may affect the accuracy of dose calculation and treatment delivery due to modulation of machine parameters, limitations in calculation algorithms, and beam and multileaf collimator (MLC) modeling [8], [9], [10], [11], [12]. The latest approach presented in the literature concerns the development of machine learning (ML) models to predict the PSQA results from plan complexity [13], [14], [15], [16], [17], [18], [19]. However, their application in clinical practice over a long period of time is yet to be reported.

In this study, we introduce a procedure to monitor the complexity and expected PSQA result of a treatment plan leveraging an in-house ML model. Our approach is based on Lean Six Sigma, a methodology introduced in the manufacturing industry to improve process quality. For our purposes, the RT planning process is under consideration, and we use quality in Lean Six Sigma terms to denote its overall reliability, consistency, and accuracy, thus encompassing dosimetric quality, plan complexity, and deliverability.

Our aim is twofold. First, to monitor outlier complexity and improve overall quality while ensuring that treatment plans still meet the desired clinical goals. Second, to reduce the risk of PSQA failure and allocate resources more efficiently, as plans with a higher likelihood of passing PSQA can undergo a streamlined QA program which does not require time-consuming measurements.

## 2 Material and methods

### 2.1 Lean Six Sigma

Lean Six Sigma combines the Lean and Six Sigma approaches. *Lean*, coined by Krafcik [20] and then defined by Womack et al. [21], focuses on eliminating non-value-added activities, or waste, within a process. In *lean healthcare*, waste refers to activities not directly benefiting the patient [22]. *Six Sigma*, a data-driven methodology introduced by Smith in 1986 [23], aims to minimize the occurrence of defects from a process and maintain a level of performance within the specification limits of ±6σ, i.e., 3.4 defects per million opportunities. Research in the late 90s began exploring Six Sigma in healthcare [24].

Lean and Six Sigma have been effectively applied in RT for treatment planning, patient setup, and working group management [25], [26], [27], [28], [29]. In this study, the Lean Six Sigma methodology was implemented in clinical practice using the five DMAIC steps: Define, Measure, Analyze, Improve, and Control. Ethical approval was not required for this study.

In the first step, the problem or opportunity for improvement was defined. A certain level of complexity in treatment plans is often required to achieve an acceptable dose distribution [6], [7], [9]. While higher complexity can improve dosimetric quality, the increased modulation of machine parameters can reduce the deliverability below tolerance. In addition, the agreement between dose calculation and PSQA measurement can be compromised due to limitations in calculation algorithms and the influence of beam and MLC modeling. Conversely, very low complexity can result in suboptimal plans where there is margin for improving dosimetric quality without affecting deliverability and calculation reliability [30]. Thus, the following problem statement was identified: “The RT optimization process might produce suboptimal plans due to extremely low or extremely high complexity”.

In the Measure phase, data were collected to quantify the current state of the process. A retrospective analysis was performed on all 28,612 volumetric modulated arc therapy (VMAT) plans (69,811 arcs) delivered in our Institute between 2013 and 2021. Details on the clinical equipment used are provided in Table S1 of the Supplementary material. For each plan, the DICOM RTPLAN was analyzed with a MATLAB script to compute ten metrics representing various aspects of plan complexity for VMAT treatments [4], [31]. While the software computed additional parameters, such as gantry speed, MLC speed, field size, and MU, in this study we focused on metrics derived from machine parameters to assess their effectiveness on large datasets. All metrics, computed per arc, are listed in Table 1.

**Table 1:** List of complexity metrics considered in this study, calculated for each arc.

	**Name**	**Description**
1	Q1 MLCGap	First quartile of the distribution of MLC gap sizes (calculated per control point)
2	Median MLCGap	Median of the distribution of MLC gap sizes (calculated per control point)
3	SAS10 [32]	Small aperture score: fraction of MLC gaps <10 mm
4	MeanTGI [33]	Mean tongue and groove index: irregularity in beam aperture shapes
5	MCS [34]	Modulation complexity score: combines segment shape and area of beam aperture
6	MITotal [35]	Modulation index for total modulation: combines MLC dynamics, gantry speed variability and dose rate variability
7	BI [36]	Beam irregularity: measures the non-circularity of the MLC aperture
8	BM [36]	Beam modulation: indicates to what extent the beam is delivered into smaller apertures (compared to the total beam area)
9	EdgeMetric [12]	Ratio of MLC side-length to aperture area
10	LT/AL [37]	Average leaf travel distance divided by the arc length

PSQA measurements and analyses were performed with the linacs’ electronic portal imaging device (EPID) using the Portal Dosimetry software (v15.6; Varian Medical Systems, Palo Alto, CA). The gamma passing rate (GPR) was computed in absolute dose with 3 %(global)/1 mm criteria, normalizing by the maximum value within the TPS dose distribution, and using a 10 % cut-off. A 90 % action limit was considered.

**Fig. 1:**
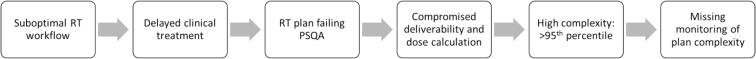
Diagram reporting the root cause of a suboptimal RT workflow, stemming from the lack of monitoring of plan complexity. Abbreviations: PSQA = patient-specific quality assurance; RT = radiotherapy.

During the Analyze stage, data were inspected to identify, validate, and select the root cause of the problem for elimination. A causal diagram, shown in Fig. 1, was outlined to characterize the source of a suboptimal RT optimization process and delivery to the patient. The initial lack of monitoring of plan complexity was recognized as root cause for a series of negative effects, which ultimately impacted the quality and timeliness of a clinical treatment.

The distributions of the complexity metrics were stratified by treatment (see Fig. S1 in the Supplementary material for the stratification statistics). To reduce the variability and improve the RT optimization process quality, outlier values of complexity were defined as below the 5th or above the 95th percentile of the distributions, i.e., either suboptimal or extremely complex plans, respectively. These thresholds identified the specification limits (*sigma level*) within which the RT optimization process should perform after the Lean Six Sigma implementation.

The aim of the Improve phase was to design, implement, and verify a solution. An ML model (xgboost) was trained to predict the GPR of an arc based on its complexity and other plan parameters, for a total of 19 numerical features. The detailed methodology and evaluation can be found in the original paper [4]. The model was trained on 5522 VMAT plans delivered from 2018 to 2022 (including data after the conclusion of the Measure phase). HyperArc (Varian) plans, fields whose size was greater than the EPID acquisition size, and incorrect/incomplete measurements were excluded. As the GPR was affected by the mechanical precision of the treatment machines, only Varian TrueBeam machines were considered.

The model, trained on the largest single-institute database of VMAT plans, achieved a mean absolute error of 2.3 %, and a sensitivity/specificity of 0.39/0.99 when predicting GPR at 3 %/1 mm with a 90 % action limit. The low sensitivity resulted from the model's objective to minimize absolute error and the highly imbalanced dataset, where 94 % of arcs had passing rates above the 90 % action limit.

A Decision Support System (DSS) tool was developed using the Eclipse Scripting Application Programming Interface (ESAPI; Varian Medical Systems, Palo Alto, CA) for the Eclipse TPS. This tool monitored the ten complexity metrics and expected PSQA result at the end of the optimization process directly in the TPS. As visual management, outlier complexities were flagged according to the historical distributions of the treatment site (see Fig. S2 in the Supplementary material).

In our previous study, we found strong correlations between similar metrics, such as Q1 MLCGap, Median MLCGap, and SAS10, which could fictitiously increase the number of outliers [4]. To prevent inflation and following the Six Sigma methodology, an arc was defined as a *defect* of the optimization process if more than five out of ten complexity metrics were outside the specification limits. Furthermore, to address the model’s low sensitivity, an arc was considered at risk of PSQA failure if more than five metrics were in the region of high complexity.

Finally, the purpose of the Control stage was to embed the solution into the RT optimization process and ensure sustainability. The DSS tool was introduced in August 2022, with follow-up results prospectively measured from September 2022 to May 2023. In a preliminary phase, the DSS tool could be used only within the TPS. Then, a *Poka Yoke* approach was implemented to sustain the solution and address the potential miss of plans at risk of PSQA failure due to the non-utilization of the tool. Poka Yoke is a Lean mechanism that helps avoid (*yokeru*) mistakes (*poka*) by preventing or drawing attention as they occur. Since November 2022, the DSS tool has run automatically at the end of each day, analyzing all approved plans.

A plan was considered at risk of PSQA failure if most of its arcs had more than five metrics in the high-complexity region or if the average predicted GPR was below a 90 % action limit. If a plan was at risk, an automatic email was sent to the planners with a report of plan complexity, expected PSQA outcome, and visualization of the impact that each feature had on the prediction of the ML model. In response, planners performed the PSQA analysis using the same criteria and, if necessary, re-optimized the RT plan.

Corrective actions were taken to increase the expected PSQA result or decrease the occurrence of defective arcs in the high complexity region. The predominant approach was to re-start the optimization with the same parameters. Other techniques included limiting MU and using the aperture shape controller of the Eclipse TPS to increase the size and decrease the complexity of the MLC aperture [30]. For plans optimized by junior planners, a senior planner supervised the results and assessed each case at risk.

### 2.2 Data analysis

Data analysis and visualizations were performed using Python 3.9.13, scipy 1.10.1, pandas 2.0.1, and seaborn 0.12.2. The Mann-Whitney test was used to compare the predicted GPR before and after re-optimization, with a significance level of 0.05.

In the following section, Q1 MLCGap, MeanTGI, and MCS metrics were considered to describe the leaf gap sizes, irregularity in beam aperture shape, and leaf/beam aperture variability, respectively. Head and neck (H&N), thorax stereotactic body radiation therapy (SBRT), abdomen SBRT, and genitourinary (GU) were selected as representative treatments spanning different anatomical sites.

## 3 Results

**Fig. 2:**
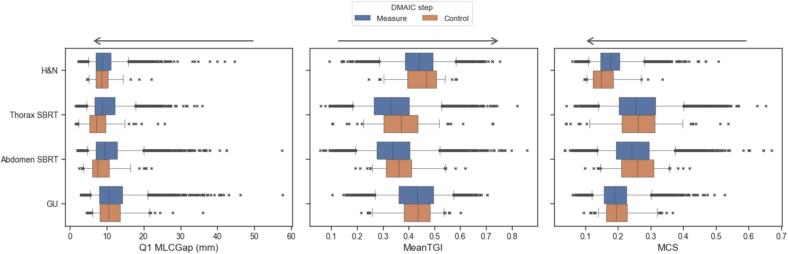
Boxplots of the complexity metrics in the Measure and Control phase, stratified by treatment, for the plans that were monitored. The crosses represent the outliers of the distributions (i.e., <5th or >95th percentile). Arrows indicate the direction of increase in complexity for each metric. A small value of Q1 MLCGap indicates smaller distances between MLC leaves pairs, resulting in increased dosimetric uncertainties due to limitations in modeling radiation transmitted at the tip of the MLC leaves. A large MeanTGI value indicates an irregular MLC aperture which can affect dose calculation accuracy due to the tongue-and-groove modeling. A small MCS value indicates a high variability in both MLC shape and area. Abbreviations: GU = genitourinary; H&N = head and neck; MCS = modulation complexity score; MeanTGI = mean tongue-and-groove index; SBRT = stereotactic body radiation therapy.

The distribution of the complexity metrics did not change abruptly between the Measure and Control phase, as shown in Fig. 2 (see Fig. S3 in the Supplementary material for the complete results). During the Control phase, the occurrences of arcs in the low and high complexity regions for Q1 MLCGap, MeanTGI, and MCS were on average 5 % and 6 %, respectively. Table 2 reports the summary statistics of these distributions.

**Table 2:** Summary statistics of the distributions of Q1 MLCGap, MeanTGI, and MCS metrics for relevant treatment sites. Measure and Control denote the stages before and after the introduction of the DSS tool in clinic. For the Control phase, the percentage variation of the statistics with respect to the Measure phase is shown. The percentage of arcs falling in the low and high complexity regions after the introduction of the tool is reported. Note that complexity increases with the metric’s value for MeanTGI, while for MCS and Q1 MLCGap the complexity decreases with the metric's value.

Treatment site	Metrics	Phase	5 %	50 %	95 %	% arcs low complexity	% arcs high complexity
H&N	Q1 MLCGap (mm)	Measure	5	9	16		
		Control	+2 %	−1 %	−7 %	5 %	5 %
	MeanTGI	Measure	0.3	0.4	0.6		
		Control	+5 %	+7 %	−6 %	2 %	2 %
	MCS	Measure	0.1	0.2	0.3		
		Control	−5 %	−16 %	−4 %	4 %	11 %

Thorax SBRT	Q1 MLCGap (mm)	Measure	5	9	18		
		Control	−50 %	−17 %	−13 %	3 %	15 %
	MeanTGI	Measure	0.2	0.3	0.5		
		Control	+21 %	+13 %	+2 %	4 %	6 %
	MCS	Measure	0.1	0.3	0.4		
		Control	−23 %	+3 %	+3 %	6 %	6 %

Abdomen SBRT	Q1 MLCGap (mm)	Measure	5	9	20		
		Control	−22 %	−18 %	−2 %	5 %	13 %
	MeanTGI	Measure	0.2	0.3	0.5		
		Control	+27 %	+7 %	+4 %	2 %	8 %
	MCS	Measure	0.1	0.2	0.4		
		Control	+8 %	+7 %	−2 %	5 %	2 %

GU	Q1 MLCGap (mm)	Measure	6	11	21		
		Control	+10 %	−1 %	+2 %	7 %	4 %
	MeanTGI	Measure	0.3	0.4	0.6		
		Control	−6 %	+1 %	−6 %	8 %	1 %
	MCS	Measure	0.1	0.2	0.3		
		Control	+12 %	+4 %	+7 %	8 %	3 %

Abbreviations: GU = genitourinary; H&N = head and neck; MCS = modulation complexity score; MeanTGI = mean tongue-and-groove index; SBRT = stereotactic body radiation therapy.

In the preliminary phase, 782 VMAT plans (1783 arcs) were analyzed directly in the TPS using the DSS tool. The calculations took less than 30 s per plan and revealed 123 (7 %) defective arcs. Among the outlier complexities, 46 % and 58 % were located in the low- and high-complexity regions, respectively. According to our criteria of complexity, 58 defective arcs were considered at risk of PSQA failure. Fig. S4 shows the comparison of the complexity metrics before and after re-optimization for all treatment sites.

**Fig. 3:**
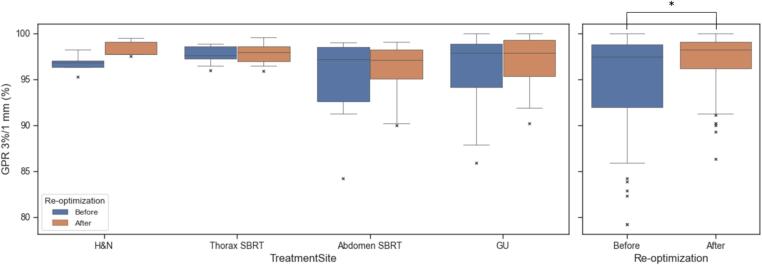
Expected GPR before and after re-optimization, stratified by representative treatment site a) and overall distribution b). Significant differences (p < 0.05) according to the Mann-Whitney test are denoted with *. Abbreviations: GPR = gamma passing rate; GU = genitourinary; H&N = head and neck; SBRT = stereotactic body radiation therapy.

The ML model integrated into the DSS tool allowed to compare multiple plans in terms of expected PSQA outcome and detect 52 arcs (3 %) with GPR <90 %. With corrective actions, the distribution of the expected GPR significantly improved (p = 0.007), with an increase in the median – [first, third] quartile – from 97.4 % [92.0, 98.8]% to 98.2 % [96.2, 99.1]%, as shown in Fig. 3. Specifically, we observed significant changes in the expected GPR for abdomen and thorax cases, from 91.1 % [88.0, 92.9]% to 97.4 % [96.4, 98.6]% (p = 0.004) and from 89.5 % [87.2, 91.4]% to 97.0 % [93.6, 98.0]% (p = 0.007), respectively (see Fig. S5 in the Supplementary material).

The Poka Yoke system analyzed 1722 VMAT plans, and 29 (1.7 %) of them were marked at risk. After the PSQA measurements, 9 out of 29 plans were found to be actual failures and were re-optimized such that they were no longer ranked at risk. Fig. S6 in the Supplementary material shows a representative report attached to the automatic email sent by the Poka Yoke system, with plan information, complexity metrics and expected PSQA outcome for each arc. We provide in Fig. S7 a representative case of false positive result.

## 4 Discussion

In this study, we presented the clinical application of ML and Lean Six Sigma to monitor outlier complexities and implement a targeted PSQA approach for VMAT plans. Our automatic tool allowed to analyze RT plans daily and take corrective actions for cases identified at risk. Nine months after its introduction, the system analyzed 1722 VMAT plans. Throughout this prospective period, the occurrences of outlier complexities remained stable and the expected median GPR (3 %/1 mm) of clinically approved plans significantly increased from 97.4 % to 98.2 %. Only 29 plans were marked at risk and nine were found to be actual failures.

The integration of artificial intelligence (AI) tools in clinical practice to streamline the RT workflow and improve patient care is rapidly rising. International Institutions are studying proposals for updating the core curriculum of medical physics experts to include AI, as well as the level of qualification needed to manage the increasing technological complexity of radiation treatments and demands on quality and risk management [38], [39]. Our system addresses the demand highlighted in the 2020 ESTRO survey for tools to evaluate complexity during and after optimization, offering a method to translate complexity into plan quality and deliverability with AI [40].

Currently, several groups are investigating methods for improving plan quality and reducing measurement-based PSQA either by using predictive models or long-term analysis of data. Marsac et al. quantified the correlation and sensitivity between complexity metrics and GPR. They identified the most appropriate metric (MCS) for reducing their PSQA workload, achieving a 30 % reduction [14]. Cavinato et al. trained a regression model for virtual PSQA of helical tomotherapy plans, estimating a 35 % workload reduction [16]. Clinical applications of ML models were reported by Wall et al. and Noblet et al. The first group presented a virtual PSQA prediction model based on complexity metrics for point dose ion chamber measurement of VMAT plans, estimating average time savings of 32.5 h per month [19]. The second group implemented a similar approach to ours, using VMAT plans, EPID measurements with 2 %/2 mm gamma criteria, and integrating into the TPS their ML model trained on complexity metrics. The authors estimated yearly savings of 140 h [15]. More broadly, Bossuyt et al. investigated the impact of EPID in-vivo dosimetry (IVD) for continuous quality improvement, noticing a gradual decrease of failed measurements over five years [17]. In another work by Mans et al., EPID IVD was used to monitor incremental changes in the RT workflow and reduce dose uncertainties [18].

Our department has introduced an independent calculation software to reduce the workload of measurement-based PSQA, with an estimated 70 % workload reduction if only the predicted failures were actually measured. In this study, Lean Six Sigma was introduced to provide complementary information to our PSQA approach. Importantly, the developed tool did not change our existing procedures and is currently utilized as an additional layer of control at the bottom of our QA pyramid (see Fig. S8 in the Supplementary material).

This study has some limitations. Despite collecting a vast number of plans, potential biases arise from process changes over the years. Notably, the TPS software in our clinic was updated in late 2019. Although plan complexity may vary with algorithm changes, we considered all historical data since 2013 to identify outlier complexity regions and leverage the large dataset collected. Plan complexity also depends on various factors such as radiation oncologists’ requirements, planners’ experience, and habits, which are difficult to control. In our recent study, we found that planner experience alone reduced plan complexity over time for a specific treatment [41]. In this work, involving many operators and parameters, the new TPS version did not disrupt the optimization process. Conversely, we excluded plans optimized using the HyperArc algorithm, which is specifically tailored to treating multiple brain metastases using a single isocenter in a single fraction, resulting in markedly different complexity compared to other brain plans. Further, we only considered a subset of complexity metrics representative of VMAT treatments, despite many described in the literature.

We selected stricter GPR criteria than AAPM TG-218, reducing the clinical relevance. With the recommended 3 %/2 mm, most cases would have been within tolerance and the ML model challenging to train to detect relevant cases, due to the extremely unbalanced data toward excellent GPRs [4]. Since VMAT PSQA rarely fails with modern TPS modeling and delivery systems, we used 3 %/1 mm with a 90 % action limit to embrace the Lean approach of continuous improvement of process quality. Although no consensus exists on stricter criteria, other authors suggest 2 %(local or global)/2 mm [42], [43].

This was a monocentric study, and our methodology is not directly transferable to other centers, as it requires the collection of new data to evaluate the state of the department optimization process. Differences in techniques, equipment, and clinical procedures may limit the proposed methodology and require further investigations.

In conclusion, this study presented a novel procedure based on the Lean Six Sigma methodology to continuously monitor plan complexity and identify cases at risk of PSQA failure. The proposed approach allowed to supervise the variability associated with the RT optimization process and implement a targeted approach for measurement-based PSQA, which directed the attention and resources of the clinical staff to rare events. As a result, we observed enhancements in the overall RT plan quality and potential benefits for patient safety.

## CRediT authorship contribution statement

**Nicola Lambri:** Conceptualization, Methodology, Software, Formal analysis, Investigation, Data curation, Writing – original draft, Visualization. **Damiano Dei:** Validation, Investigation, Data curation, Writing – review & editing. **Giulia Goretti:** Resources, Writing – review & editing, Supervision. **Leonardo Crespi:** Software, Formal analysis, Data curation, Writing – review & editing, Visualization. **Ricardo Coimbra Brioso:** Software, Formal analysis, Data curation, Writing – review & editing, Visualization. **Marco Pelizzoli:** Validation, Data curation, Writing – review & editing, Visualization. **Sara Parabicoli:** Validation, Data curation, Writing – original draft, Writing – review & editing, Visualization. **Andrea Bresolin:** Software, Data curation, Writing – review & editing. **Pasqualina Gallo:** Validation, Data curation, Writing – review & editing. **Francesco La Fauci:** Validation, Data curation, Writing – review & editing. **Francesca Lobefalo:** Validation, Data curation, Writing – review & editing. **Lucia Paganini:** Validation, Data curation, Writing – review & editing. **Giacomo Reggiori:** Resources, Writing – review & editing, Supervision. **Daniele Loiacono:** Resources, Writing – review & editing, Supervision. **Ciro Franzese:** Resources, Writing – review & editing, Supervision. **Stefano Tomatis:** Resources, Writing – review & editing, Supervision. **Marta Scorsetti:** Resources, Writing – review & editing, Supervision. **Pietro Mancosu:** Methodology, Resources, Writing – review & editing, Supervision, Project administration, Funding acquisition.

## Declaration of Competing Interest

Pietro Mancosu is an Editorial Board Member/Editor-in-Chief/Associate Editor/Guest Editor for Physics and Imaging in Radiation Oncology and was not involved in the editorial review or the decision to publish this article.
