# Supplementary material for: Machine learning and lean six sigma for targeted patient-specific quality assurance of volumetric modulated arc therapy plans
Source: Phys Imaging Radiat Oncol. 2024 Aug 9;31:100617. doi: 10.1016/j.phro.2024.100617 (PMC11367262; doi:10.1016/j.phro.2024.100617)
Supplement: Supplementary Data 1 [file mmc1.pdf]

Supplementary material

Table S1: Composition of the dataset. All hardware and software considered is from Varian. The dose grid size values are ordered by frequency. All PSQA measurements in this study were analyzed with Portal Dosimetry, which was introduced with Eclipse 15.6. The dose reporting mode of AcurosXB is dose-to-medium.

	**Machine**	**MLC**	**Activity time**	**Num. of arcs**
**Treatment Unit**	UNIQUE	Millennium	2012 - 2020	11831
	DHX	Millennium	2005 - 2023	12421
	TrueBeamSN1015	Millennium	2010 - operating	12663
	TrueBeamSN4791	Millennium	2021 - operating	1459
	TrueBeamSTX_1280	Millennium High Definition	2012 - operating	14110
	EDGE_2005	Millennium High Definition	2014 - operating	17327
	**Version**	**Optimization algorithm**	**Dose calculation algorithm**	**Grid size (cm)**
**TPS Eclipse**	11.0 up to Nov 2019 (52963 arcs)	PRO (10.0.28)	AAA 10.0.28	0.25, 0.15, 0.20, 0.10
	15.6 since Nov 2019 (16848 arcs)	PO (15.6.06)	AcurosXB 15.6.06 (15474 arcs)<br>AAA 15.06.06 (1374 arcs)	0.25, 0.125
	**Method**	**Algorithm (version)**	**Portal Imager**	**Calibration**
**PSQA Portal Dosimetry**	EPID	PDIP (15.6)	aS1200<br>aS1000 (TrueBeamSN1015 and TrueBeamSTX_1280 up to 2019)	Dose normalization 10x10 cm^2 (monthly)

Abbreviations: AAA = analytical anisotropic algorithm; EPID = electronic portal imaging device; MLC = multileaf collimator; PDIP = portal dose image prediction; PSQA = patient-specific quality assurance.

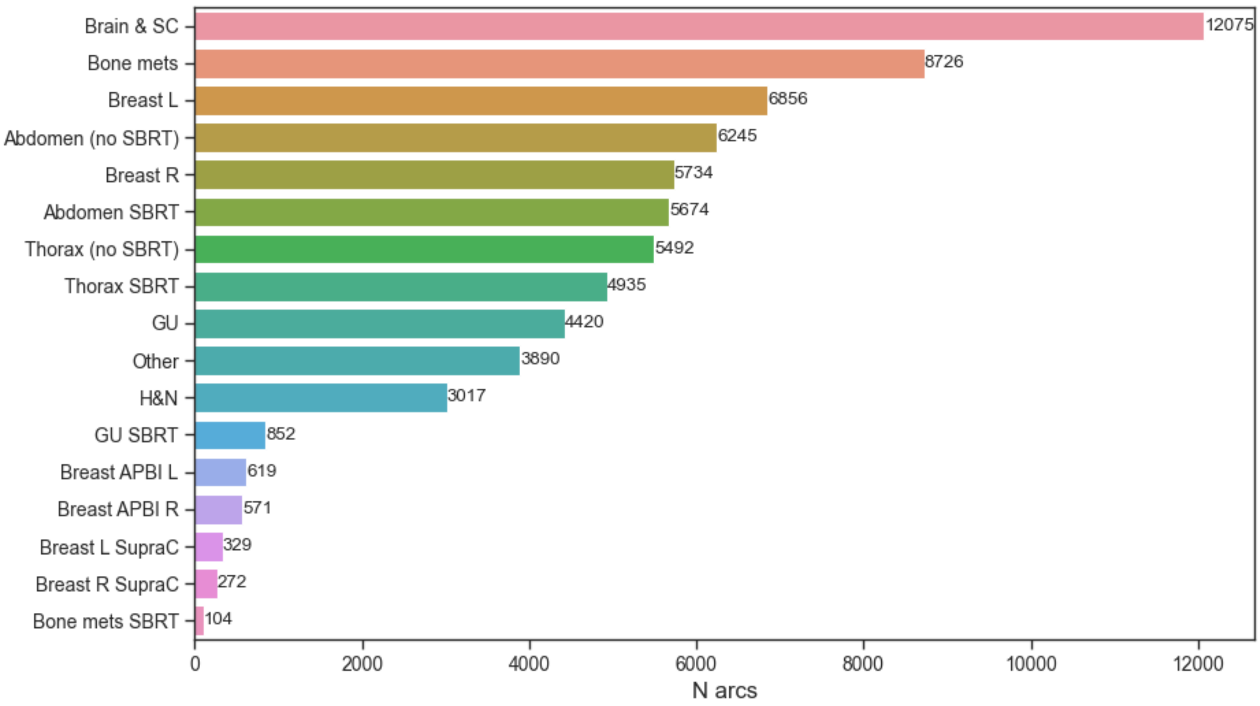

Figure S1: Number of arcs considered for the retrospective analysis, stratified by treatment site. *Abbreviations: APBI = accelerated partial breast irradiation; Bone mets = bone metastases; Brain & SC = brain & spinal cord; GU = genitourinary; H&N = head & neck; SBRT = stereotactic body radiation therapy; SupraC = supraclavicular.*

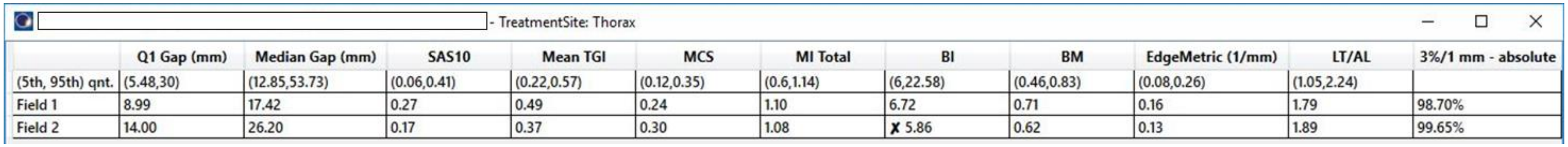

- TreatmentSite: Thorax

	Q1 Gap (mm)	Median Gap (mm)	SAS10	Mean TGI	MCS	MI Total	BI	BM	EdgeMetric (1/mm)	LT/AL	3%/1 mm - absolute
(5th, 95th) qnt.	(5.48,30)	(12.85,53.73)	(0.06,0.41)	(0.22,0.57)	(0.12,0.35)	(0.6,1.14)	(6,22.58)	(0.46,0.83)	(0.08,0.26)	(1.05,2.24)	
Field 1	8.99	17.42	0.27	0.49	0.24	1.10	6.72	0.71	0.16	1.79	98.70%
Field 2	14.00	26.20	0.17	0.37	0.30	1.08	✗ 5.86	0.62	0.13	1.89	99.65%

Figure S2: Output of the DSS tool presented to the user for the plan currently opened in the TPS. The table contains ten complexity metrics and expected PSQA outcome for each arc. As visual management, outlier complexities are flagged according to the historical distributions of the treatment site.

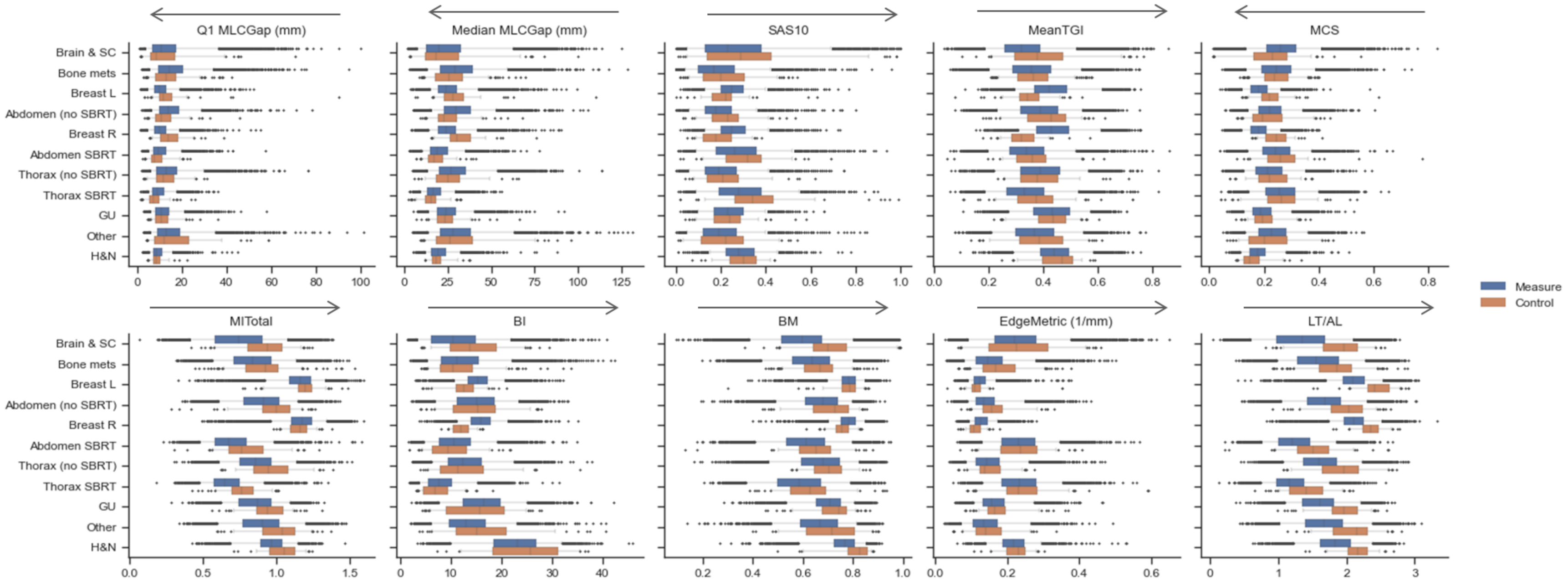

Figure S3: Boxplots of the complexity metrics in the Measure and Control phase, stratified by treatment. Treatments with more than 40 arcs in the Control phase are reported. The crosses represent the outliers of the distributions (i.e., <5th or >95th percentile). Arrows indicate the direction of increase in complexity for each metric. *Abbreviations: Bone mets = bone metastases; Brain & SC = brain & spinal cord; GU = genitourinary; H&N = head & neck; SBRT = stereotactic body radiation therapy.*

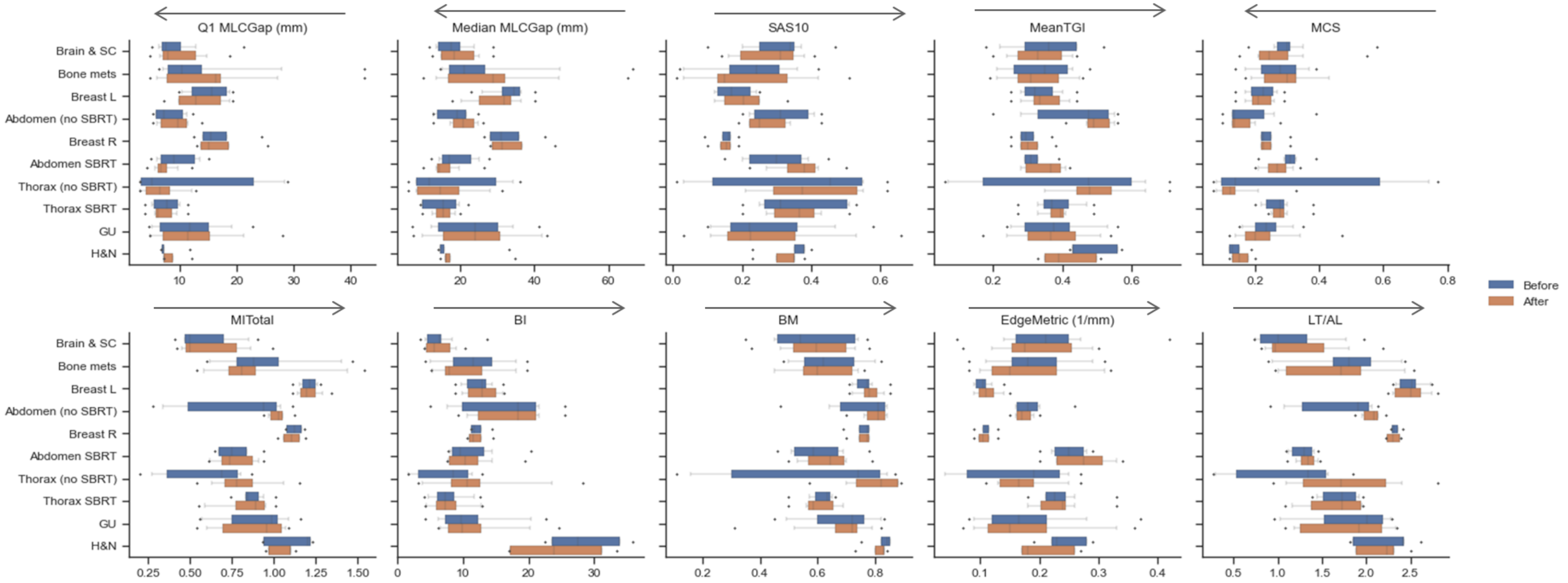

Figure S4: Boxplots of the complexity metrics for different iterations of optimization, stratified by treatment. Treatments with more than 40 arcs in the Control phase are reported. The crosses represent the outliers of the distributions (i.e., <5th or >95th percentile). Arrows indicate the direction of increase in complexity for each metric. *Abbreviations: Bone mets = bone metastases; Brain & SC = brain & spinal cord; GU = genitourinary; H&N = head & neck; SBRT = stereotactic body radiation therapy.*

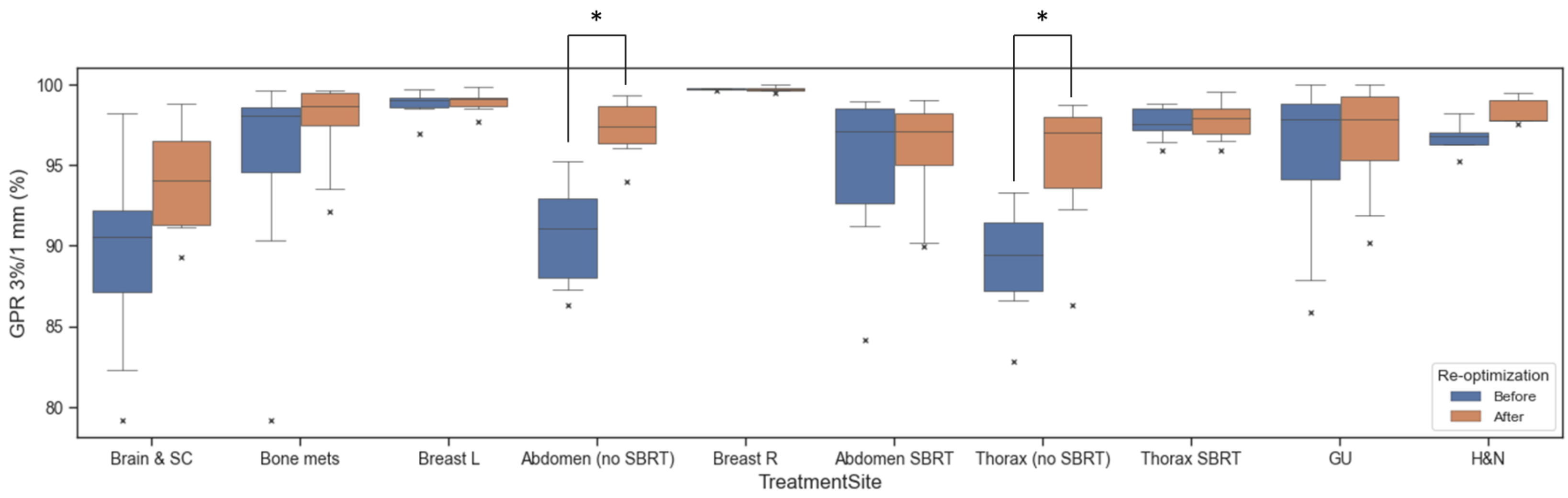

Figure S5: Expected GPR before and after re-optimization, stratified by treatment site. Treatments with more than 40 arcs in the Control phase are reported. Significant differences ($p < 0.05$) according to the Mann-Whitney test are denoted with *. *Abbreviations: Bone mets = bone metastases; Brain & SC = brain & spinal cord; GU = genitourinary; H&N = head & neck; SBRT = stereotactic body radiation therapy.*

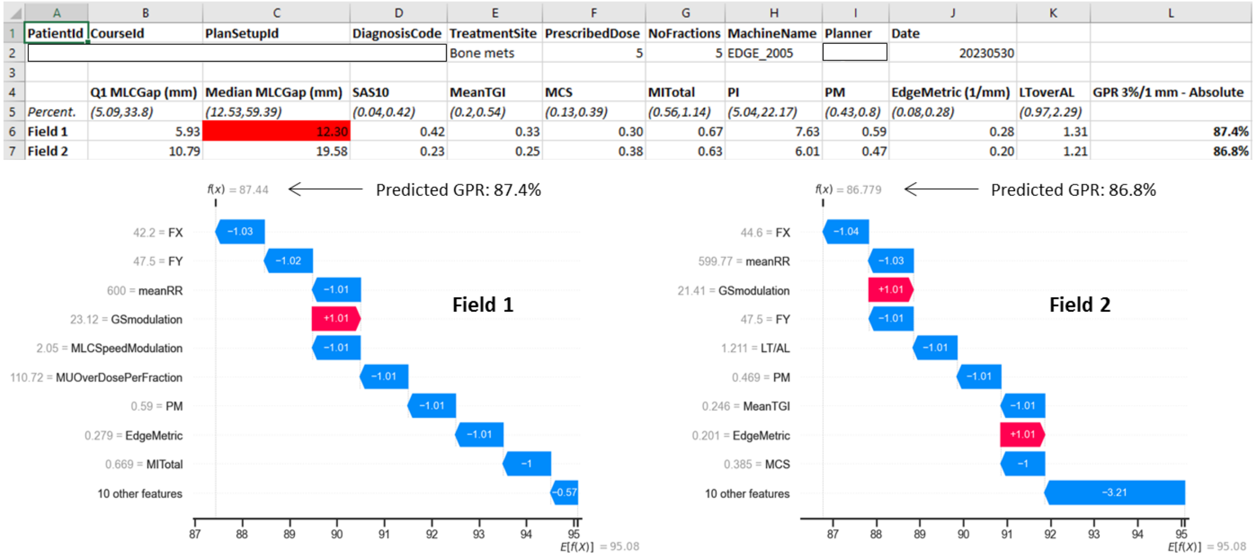

	A	B	C	D	E	F	G	H	I	J	K	L
1	**PatientId**	**CourseId**	**PlanSetupId**	**DiagnosisCode**	**TreatmentSite**	**PrescribedDose**	**NoFractions**	**MachineName**	**Planner**	**Date**		
2					Bone mets	5	5	EDGE_2005		20230530		
3												
4		**Q1 MLCGap (mm)**	**Median MLCGap (mm)**	**SAS10**	**MeanTGI**	**MCS**	**MITotal**	**PI**	**PM**	**EdgeMetric (1/mm)**	**LToverAL**	**GPR 3%/1 mm - Absolute**
5	*Percent.*	*(5.09,33.8)*	*(12.53,59.39)*	*(0.04,0.42)*	*(0.2,0.54)*	*(0.13,0.39)*	*(0.56,1.14)*	*(5.04,22.17)*	*(0.43,0.8)*	*(0.08,0.28)*	*(0.97,2.29)*	
6	**Field 1**	5.93	12.30	0.42	0.33	0.30	0.67	7.63	0.59	0.28	1.31	**87.4%**
7	**Field 2**	10.79	19.58	0.23	0.25	0.38	0.63	6.01	0.47	0.20	1.21	**86.8%**

Figure S6: Automatic report sent by email in case a plan is considered at risk of PSQA failure and needs further attention from the planner. The report contains the plan information, complexity metrics, and expected PSQA outcome. For explainability purposes, the report also contains a visual representation showing the impact that each feature (complexity metrics and plan parameters) had on the prediction of the ML model. For instance, the plot on the left shows that, starting from a baseline value of 95.1% (light gray at the bottom-right corner) the feature value FX=42.2 mm contributes "-1.03%" at lowering the predicted GPR. The features are ranked from top to bottom by contribution importance. The sum of all the contributions of each feature value brings the model prediction to 87.4%. The baseline value is inferred by SHAP by inspecting the model's tree structure. *Abbreviations: GPR = gamma passing rate; ML = machine learning; PSQA = patient-specific quality assurance.*

	A	B	C	D	E	F	G	H	I	J	K	L
1	PatientId	CourseId	PlanSetupId	DiagnosisCode	TreatmentSite	PrescribedDose	NoFractions	MachineName	Planner	Date		
2					Abdomen SBRT	12	4	TrueBeamSTX_1280		20230125		
3												
4		Q1 MLCGap (mm)	Median MLCGap (mm)	SAS10	MeanTGI	MCS	MITotal	PI	PM	EdgeMetric (1/mm)	LToverAL	GPR 3%/1 mm - Absolute
5	*Percent.*	*(4.69,19.98)*	*(9.57,35.31)*	*(0.09,0.52)*	*(0.19,0.52)*	*(0.14,0.38)*	*(0.47,0.99)*	*(4.49,19.34)*	*(0.41,0.8)*	*(0.13,0.35)*	*(0.75,1.86)*	
6	**Field 1**	23.53	27.54	0.02	0.05	0.78	0.39	1.73	0.13	0.07	0.27	**90.7%**
7	**Field 2**	19.10	22.85	0.03	0.07	0.55	0.30	1.85	0.17	0.10	0.21	**88.5%**

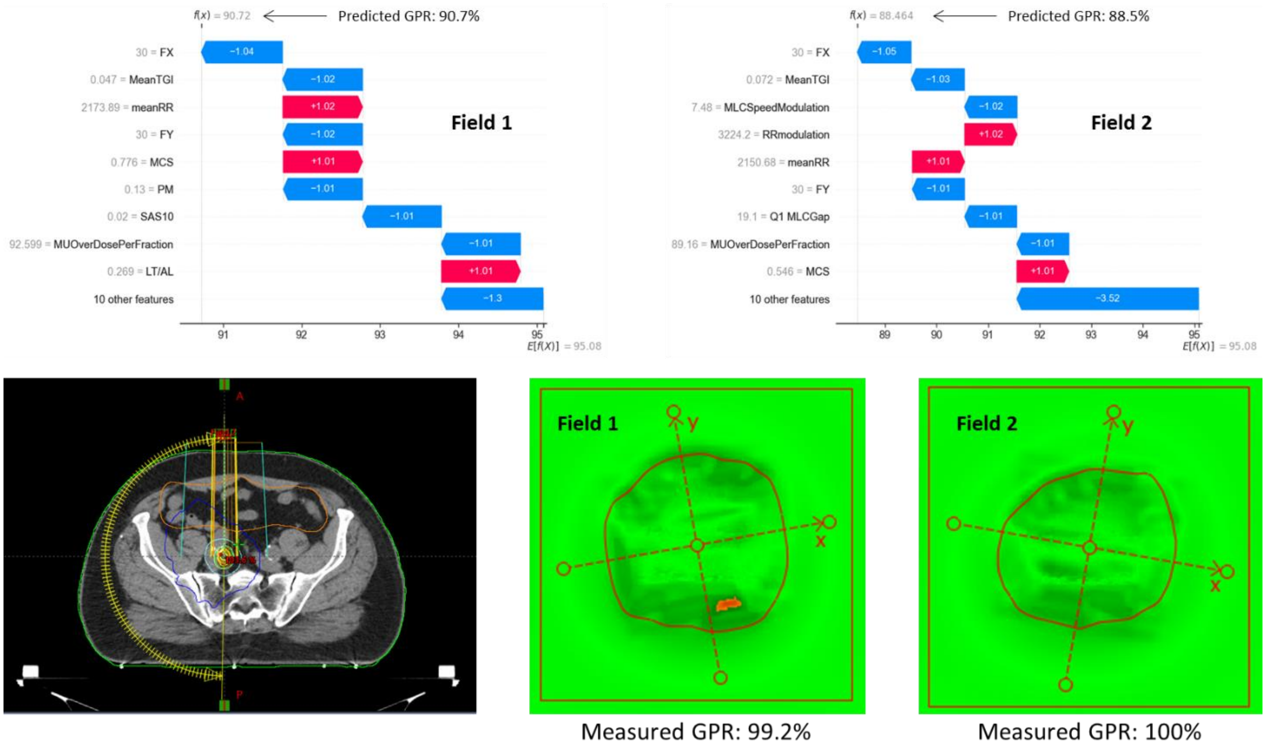

Figure S7: Extreme case of abdomen SBRT with 8 and 9 outlier complexity metrics (low complexity) and expected PSQA failure. The measurement revealed a GPR of nearly 100%. The first factor which drove the model decision to a low GPR was the small field aperture (FX=30 mm) due to the small planning target volume (3.84 cm^3). The arrows in the plots represent the positive (red) or negative (blue) contribution of each feature to the final predicted value of GPR. We hypothesized two concurring factors that could explain this discrepancy. On the one hand, the small field likely caused a lack of lateral electronic equilibrium, forcing the EPID to operate at the limits of its capabilities and leading to an optimistically high GPR. On the other hand, the calculation grid was smaller than standard treatments, i.e., 1.25 mm vs. 2.5 mm. Thus, the Portal Dosimetry algorithm utilized a finer sampling of the fluence, providing a more accurate calculation of the GPR. After a thorough evaluation, the case was considered clinically acceptable. *Abbreviations: EPID = electronic portal imaging device; GPR = gamma passing rate; ML = machine learning; PSQA = patient-specific quality assurance; SBRT = stereotactic body radiation therapy.*

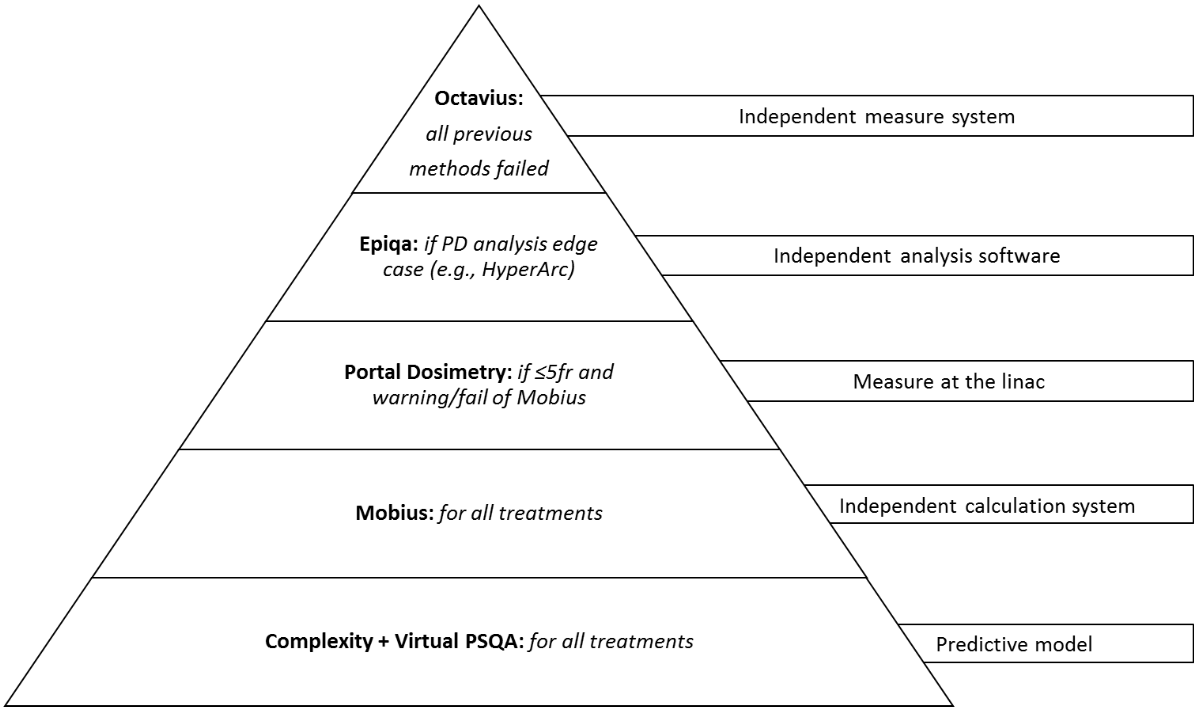

Figure S8: The PSQA program adopted in our department. After the Lean Six Sigma implementation, an extra layer of control for monitoring complexity and expected PSQA was introduced. *Abbreviations: PD = Portal Dosimetry; PSQA = patient-specific quality assurance.*
